# Renal outcomes of idiopathic and atypical membranous nephropathy in adult Chinese patients: a single center retrospective cohort study

**DOI:** 10.1186/s12882-021-02348-4

**Published:** 2021-04-22

**Authors:** Zhenbin Jiang, Meishun Cai, Bao Dong, Yu Yan, Yina Wang, Xin Li, Chunying Shao, Li Zuo

**Affiliations:** Department of Nephrology, Peking University People’s Hospital, 11 Xizhimennan Street, Xicheng District, 100044 Beijing, China

**Keywords:** Idiopathic membranous nephropathy, Atypical membranous nephropathy, Worsening renal function

## Abstract

**Background:**

Membranous nephropathy (MN) is mainly classified into idiopathic MN (iMN) and secondary MN in etiology. In recent years, a new kind of membranous nephropathy, atypical membranous nephropathy (aMN) which shows “full house” in immunofluorescence but without definite etiology was paid more attention. In a single center cohort, the renal outcomes of iMN and aMN were compared.

**Methods:**

iMN and aMN patients were selected from renal pathology databank from January 2006 to December 2015. Patients’ demographics, laboratory values, induction regimens and patients’ responses were recorded. Specially, creatinine, eGFR, albumin and 24 h urinary protein excretion were recorded at 6th month after the induction of immunosuppressive (IS) treatment and at the end of follow up. Complete proteinuria remission was defined as urinary protein < 0.3 g/d, partial proteinuria remission was defined as urinary protein between 0.3 g/d ~ 3.5 g/d and decreased > 50 % from the baseline. The primary outcome was worsening renal function, defined as a 30 % or more decrease in eGFR or end-stage renal disease (eGFR < 15ml/min/1.73m^2^). COX proportional hazard models were used to test if aMN was a risk factor of worsening renal function compared with iMN.

**Results:**

There were 298 patients diagnosed with MN and followed in our center for 1 year or more, including 145 iMN patients with an average follow-up time of 4.5 ± 2.6 years, and 153 aMN patients with 4.1 ± 2.0 years (*p* = 0.109). The average age of iMN patients was older than aMN patients (56.1 ± 12.2 versus 47.2 ± 16.2 years old, *p* < 0.001). There were 99 iMN patients and 105 aMN patients with nephrotic range proteinuria and without previous immunosuppressive treatment. 93 (93.9 %) and 95 (90.5 %) patients underwent immunosuppressive treatment in iMN and aMN group, and there was no significant difference of the overall proteinuria remission rates at 6th month (59.1 % vs. 52.0 %, *p* = 0.334) and endpoint (73.7 % vs. 69.5 %, *p* = 0.505) between the two groups. 25 (25.3 %) patients in iMN group and 21 (20.0 %) patients in aMN group reached primary endpoint (X^2^ = 0.056, *p* = 0.812). Multivariate COX regression showed that after demographics, baseline laboratory values and remission status at 6th month were adjusted, aMN group had similar renal outcome compared with iMN group, the HR of primary outcome was 0.735 (95 % CI 0.360 ~ 1.503, *p* = 0.399).

**Conclusions:**

The proteinuria remission rates and renal outcomes were similar in iMN and aMN patients after covariables were adjusted.

## Background

Membranous nephropathy (MN) remains a leading cause of nephrotic syndrome in adults[1], and the incidence rate of MN is increasing due to the environmental pollution and other factors[2]. MN is a common etiology of end-stage renal disease (ESRD), progressive loss of renal function occurs in 60 % of untreated patients, and about 35 % of these patients develop ESRD within 10 years[3–5], only 10 % or less will develop ESRD over the subsequent 10 years with proper management[6]. MN can be classified into idiopathic membranous nephropathy (iMN) without identified causes and secondary membranous nephropathy (sMN) attributed to immune diseases, malignancy, infections, or some other causes. The most important process in the diagnosis of MN is to determine it as idiopathic or secondary according to the clinical manifestations, laboratory examination and renal biopsy, which in turn guides the treatment and evaluating prognosis.

In recent years, a new kind of MN was increasing in China, which showed cells proliferation, electron dense deposits deposited in multisite, and most of patients showed “full house” in immunofluorescence, that is IgG, IgA, IgM, C3, C1q positive, but without definite etiology such as systemic lupus erythematosus (SLE), hepatitis B virus (HBV) infection, or some other known causes in clinical, which caught people’s eyes gradually. This category of MN was currently temporarily diagnosed as atypical membranous nephropathy (aMN)[7], lupus-like membranous nephropathy[7], or “full house” membranous nephropathy[8], some scholars considered that it was a new kind of MN, and its baseline characteristics and disease prognosis were between iMN and sMN patients[9], but no final conclusion had yet been reached on this matter. Our previous study had compared the clinical and pathological characteristics of iMN and aMN patients, discovered the mainly clinical manifestation of these two groups was nephrotic syndrome (61.5 % in iMN group vs. 58.4 % in aMN group), but there were more patients accompanied with nephritis syndrome in aMN group than iMN group (17.1 % vs. 6.1 %, P < 0.001), there was slight difference on laboratory examination between the two groups, serum anti-phospholipase A2 receptor (PLA2R) antibody could not distinguish aMN from iMN[7]. This study summarized the characteristics of response to treatment and renal function outcomes between the iMN and aMN patients.

## Methods

### Study participants

The study participants were similar to our previous studies and were briefly described as follows[7]. We collected all the patients diagnosed as membranous nephropathy by clinical manifestation and renal biopsy in Peking University People’s Hospital from January 2006 to December 2015 for this study. Inclusion criteria: (1) iMN group: MN patients with unknown etiology and characterized glomerular lesions of only immune complex deposited under the epithelial and thickening glomerular basement membrane. (2) aMN group: MN patients with unknown etiology in clinical, negative for antinuclear antibodies, anti dsDNA antibody, Hepatitis B surface antigen, e antigen, e antibody, and core antibody, but the renal pathology of them showed mesangial cells and matrix proliferation, immune complex and electron dense deposits deposited in subepithelial, subendothelial, and the basement membrane, and most immunofluorescence results of them showed “full house”, that is IgG, IgA, IgM, C3, C1q positive, in addition to the glomerular basement membrane lesions. Exclusion criteria: MN patients lacking immunofluorescence data and negative for IgG were excluded, MN secondary to some known causes, besides, MN accompanied with other pathological patterns, such as diabetic nephropathy, IgA nephropathy, and so on, were excluded, patients developed SLE during follow-up were ruled out.

### Baseline data collection

The clinical and laboratory examination data of selected patients were recordedat the time of renal biopsy: (1) Demographics: gender, age, prodromic infection, blood pressure, smoking status (smoking 1 cigarette a day or more, continuous or accumulative for 6 months). (2) Laboratory values: (i) Kidney damage indicators: microscopic haematuria, 24 h urinary protein excretion (24hUPE), serum creatinine, urea, uric acid (UA), eGFR level (calculated by CKD-EPI formula[10]), serum albumin, blood lipid; (ii) Immunological indicators: Serum complement (C3 and C4), serum IgG, IgA and IgM; (iii) Detection of serum anti-PLA2R antibody: ELISA method was used to detect the antibody levels of PLA2R in patients’ serum, The Anti-PLA2R ELISA (IgG) kits were purchased from EUROIMMUN Mediziniche Labordiagnostika AG.

### Follow‐up data collection

Induction regimens immediately after renal pathological diagnosis and laboratory values, which include 24hUPE, serum creatinine, eGFR level, and serum albumin during follow-up were recorded, and the indicators above at 6th month after the induction of immunosuppressive (IS) treatment were used for prognostic analysis. Besides, response of 24hUPE to treatment was recorded, complete proteinuria remission was defined as urinary protein < 0.3 g/d, partial proteinuria remission was defined as urinary protein between 0.3 g/d-3.5 g/d and decreased > 50 % from the baseline. The overall remission means partial and complete remission.

### Outcomes

The primary outcome was worsening of the renal function, defined as a 30 % or more decrease in eGFR or ESRD (eGFR < 15ml/min/1.73m^2^). The endpoints were the occurrence of primary outcomes or the observation time up to June 2020.

### Ethical committee

Our study passed ethical review by the ethics committee of Peking University People’s Hospital (2017PHB141-01). Informed consent of the patients was not obtained because of the laboratory values used in our study were consulted from routine examination documents and analyzed retrospectively.

### Statistical analysis

SPSS 22.0 statistical software was used for data analysis. The measurement data accorded with normal distribution were presented as mean ± SD and differences between two groups were compared using t-test. The non-normally distributed data were presented as medians (25th, 75th percentiles) and differences between two groups were compared using non-parametric Mann-Whitney U test. Categorical variables were compared using chi-square test or Fisher exact test. Our study mainly compared the difference of renal outcomes between iMN and aMN patients, the outcomes’ times to worsening renal function were analysed using Kaplan–Meier curves and log-rank test. COX proportional hazard regression was performed to compare the rate of primary endpoint between aMN and iMN after baseline demographics, laboratory values and proteinuria remission status at 6th month were adjusted, hazard ratios (HR) and 95 % confidential intervals (95 % CI) were calculated. The adjusting variables were selected into the multivariate COX regression model (Enter selection; *p* < 0.20 criterion for variable retention) based on the univariate COX regression analysis and clinical judgements. Two models were established. Model 1 was designed to test whether aMN was a risk factor for worsening renal function, which included classification of diseases, proteinuria remission status at 6th month and baseline characteristics, and model 2 was designed to identify differences in the effect of proteinuria remission status at 6th month on the primary endpoint between the two groups, which included the proteinuria remission status at 6th month of two groups and baseline characteristics. p < 0.05 were considered statistically significant, p < 0.01 were considered notably statistically significant.

## Results

### Study participants and the baseline characteristics

From January 2006 to December 2015, there were 3210 cases of renal biopsy in our center and membranous nephropathy accounted for 820(25.5 %) cases of total, including 351(10.9 %) iMN patients, 364(11.3 %) aMN patients and 105(3.3 %) sMN patients. There were 298 patients who met the inclusion criteria and followed up in our center for 1 year or more, including 145 patients in iMN group with an average follow-up time of 4.5 ± 2.6 years, and 153 patients in aMN group with 4.1 ± 2.0 years, with no statistical difference in follow-up time between the two groups (*p* = 0.109). Demographics and baseline laboratory values were shown in Table 1. The average age of iMN patients was significantly older than aMN patients (56.1 ± 12.2 versus 47.2 ± 16.2 years old, *p* < 0.001). The baseline eGFR level of iMN patients was lower than aMN patients (90.59 ± 20.71 versus 97.75 ± 23.83 ml/min/1.73m^2^, *p* = 0.006), the blood IgG level of iMN patients was higher than aMN patients (7.70 ± 4.14 versus 6.85 ± 2.93 g/L, *p* = 0.047), while there were no significant differences in 24hUPE, blood lipid and other immunological indicators between the two groups. The antibody levels of PLA2R were detected in 58 iMN patients and 82 aMN patients, and there was no different of concentration between the two groups.

**Table 1:** Demographics and baseline laboratory values of iMN and aMN patients

	iMN group (*n* = 145)	aMN group (*n* = 153)	*p* value
**Demographics**			
Gender (male %)	78(53.8 %)	89(56.3 %)	0.658
Age (years old)	56.1 ± 12.2	47.2 ± 16.2	< 0.001
Prodromic infection(cases)	11(7.6 %)	15(9.5 %)	0.554
Systolic pressure (mmHg)	132.4 ± 19.0	134.7 ± 18.3	0.121
Diastolic pressure (mmHg)	81.1 ± 11.4	83.2 ± 10.7	0.089
Smoking rate (%)	38(26.2 %)	50(31.6 %)	0.298
**Laboratory values**			
Microscopic hematuria(/uL)	42.0(16.7,105.3)	53.3(21.8,109.1)	0.292
24hUPE(g/24 h)	5.10(2.79,8.37)	5.50(2.96,8.88)	0.997
Urea(mmol/L)	5.55 ± 2.35	5.40 ± 2.45	0.589
Serum creatinine(umol/L)	74.27 ± 26.92	74.74 ± 28.30	0.882
eGFR(ml/min/1.73m^2^)	90.59 ± 20.71	97.75 ± 23.83	0.006
Uric acid(mmol/L)	354.98 ± 99.16	365.99 ± 105.50	0.355
Albumin(g/L)	27.77 ± 6.38	26.70 ± 7.15	0.173
Triglyceride (mmol/L)	2.73 ± 2.34	3.04 ± 2.38	0.263
Cholesterol (mmol/L)	7.69 ± 2.42	7.21 ± 2.43	0.095
LDL-C (mmol/L)	4.70 ± 1.97	4.44 ± 2.29	0.313
HDL-C (mmol/L)	1.32 ± 0.45	1.50 ± 2.37	0.393
Blood IgA(g/L)	2.21 ± 1.04	2.36 ± 1.11	0.225
Blood IgG(g/L)	7.70 ± 4.14	6.85 ± 2.93	0.047
Blood IgM(g/L)	1.25 ± 0.71	1.20 ± 0.65	0.601
Blood C3(g/L)	1.10 ± 0.25	1.07 ± 0.24	0.314
Blood C4(g/L)	0.28 ± 0.12	0.28 ± 0.11	0.598
Anti-PLA2R antibody (RU/ml)^a^	11.0(1.0,58.5)	35.0(1.0,96.0)	0.061

*LDL-C* low density lipoprotein cholesterol; *HDL-C* high density lipoprotein cholesterol; *24hUPE *24 h urinary protein excretion. ^a^The antibody levels of PLA2R were detected in 58 iMN patients and 82 aMN patients

### Renal pathology of iMN and aMN patients

**Fig. 1:**
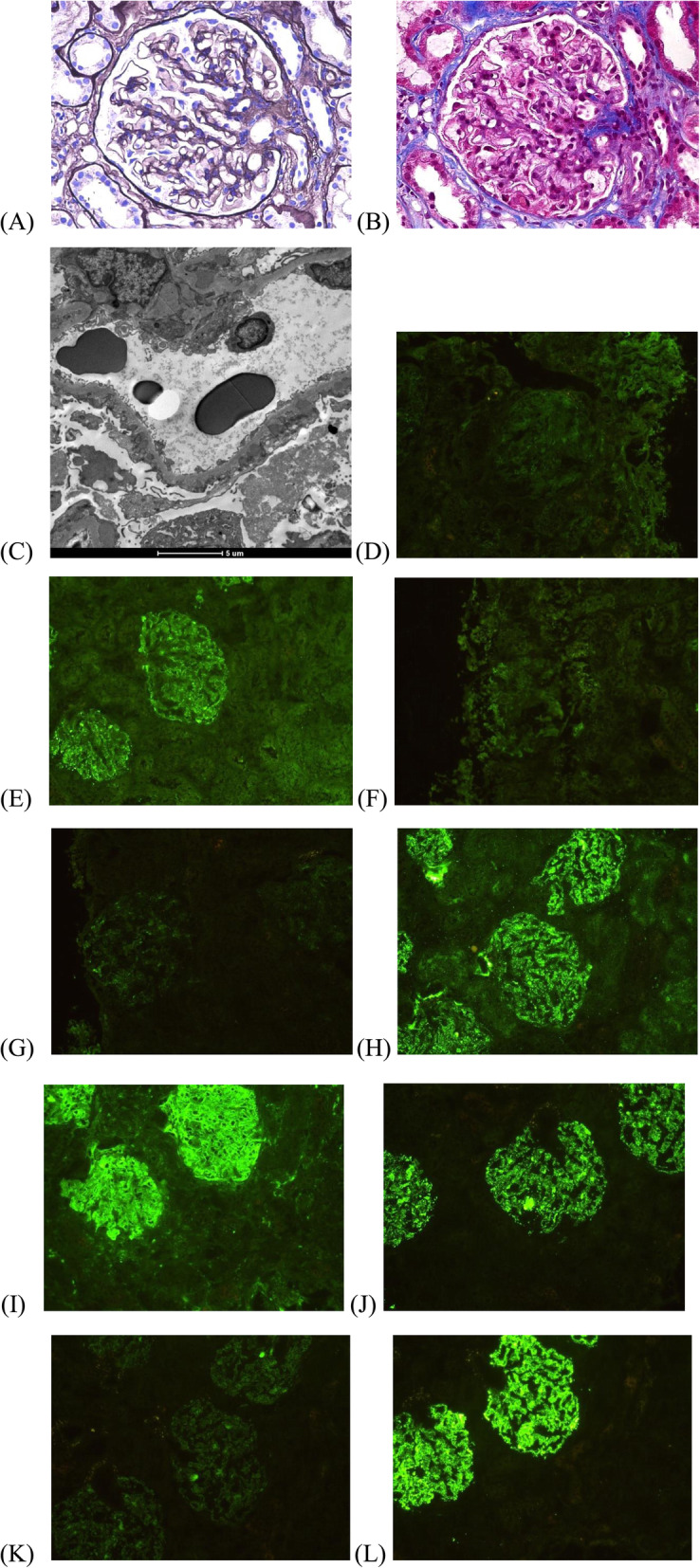
Renal pathology of one aMN patient. **a**, **b** Optical microscope image demonstrates mesangial cells and matrix proliferation, immune complex deposited in multiple locations (**a**: periodic acid-silver metheramine, PASM. **b**: Masson staining, high power field); **c** The electron micrograph demonstrates the dense deposits in subepithelial, subendothelial, and the basement membrane; Immunofluorescence test: **d** IgA(++); **e** IgG(+++); **f** IgM(++); **g** C1q(++); **h** C3(+++); **i** FRA(+++); **j** IgG1(+++); **k** IgG2(++); (not shown)IgG3(-); **l **IgG4(+++).

The renal pathology of aMN showed mesangial cells and matrix proliferation, immune complex deposited in multiple locations, the electron micrograph demonstrates the dense deposits in subepithelial, subendothelial, and the basement membrane (Fig. 1). As shown in Table 2, the most renal immunofluorescence test of aMN patients characterized by “full house”, with significant different in positive rate of IgA, IgM, C1q, C3 and FRA between iMN and aMN patients but IgG. A total of 39 iMN and 102 aMN patients had completed the IgG subtype test of renal tissue, the highest positive rate was IgG4 (94.9 %) in iMN group, and the lowest was IgG3 (2.7 %); but in aMN group, the highest positive rate was IgG1 (98.0 %), followed with IgG4 (94.1 %), with significant different of positive rate of IgG1, IgG2 between two groups but IgG3 and IgG4.

**Table 2:** The immunofluorescence test of renal biopsy in iMN and aMN patients

Characteristics	iMN group (*n* = 145)	aMN group (*n* = 153)	*p* value
**Immunofluorescence score, mean/median (range)**			
IgA	0.12/0(0,0)	1.52/2(1,2)	< 0.001
IgG	2.46/2(2,3)	2.54/3(2,3)	0.181
IgM	0.70/0(0,2)	1.49/2(1,2)	< 0.001
C1q	0.15/0(0,0)	1.59/2(1,2)	< 0.001
C3	1.82/2(2,2)	2.18/2(2,3)	< 0.001
FRA	0.31/0(0,0)	0.62/0(0,2)	0.004
IgG1	1.95/2(2,3)	2.26/2(2,3)	0.074
IgG2	0.15/0(0,0)	1.15/1(0,2)	< 0.001
IgG3	0.05/0(0,0)	0.25/0(0,0)	0.072
IgG4	2.56/3(2,3)	2.61/3(2,3)	0.688
**Positive patient, n (%)**			
IgA	15 (10.3 %)	126 (82.4 %)	< 0.001
IgG	145 (100 %)	153 (100 %)	/
IgM	60 (41.4 %)	126 (82.4 %)	< 0.001
C1q	16 (11.0 %)	133 (86.9 %)	< 0.001
C3	135 (93.1 %)	150 (98.0 %)	0.047
FRA	30 (20.7 %)	51 (33.3 %)	0.014
IgG1	34/39(87.2 %)	100/102(98.0 %)	0.018
IgG2	3/39(7.7 %)	72/102(70.6 %)	< 0.001
IgG3	1/39(2.7 %)	13/102(12.7 %)	0.112
IgG4	37/39(94.9 %)	96/102(94.1 %)	1.000

### Induction regimen and remission rate of iMN and aMN

There were 99 iMN patients and 105 aMN patients with nephrotic range proteinuria and without previous IS treatment, and 24hUTP were similar in two groups [7.20(4.96, 9.95) vs. 6.75(5.42, 10.50), *p* = 0.823]. There were 93 (93.9 %) and 95 (90.5 %) who underwent IS treatment in iMN and aMN group, respectively (Table 3). For iMN patients, 80 of them (80.8 %) received glucocorticoids + cyclophosphamide (GC + CTX) as induction, 6 of them (6.1 %) received glucocorticoids + calcineurin inhibitors (GC + CNIs) as induction. For aMN patients, 77 (73.3 %) received GC + CTX, 13 (12.4 %) received GC + CNIs as induction.

**Table 3:** Induction regimens and proteinuria remission rate of iMN and aMN patients

	iMN group (*n* = 99)	aMN group (*n* = 105)	*p* value
**Induction regimens**			0.284
Supportive care only	6(6.1 %)	10(9.5 %)	
GC + CTX	80(80.8 %)	77(73.3 %)	
GC + CNIs	6(6.1 %)	13(12.4 %)	
Other ISTs	7(7.0 %)	5(4.8 %)	
**Proteinuria remission rates at 6th month**			
24hUPE(g/24 h)	1.62(0.58,6.00)	2.60(1.15,6.23)	0.823
Complete remission	18/88(20.5 %)	10/98(10.2 %)	0.051
Overall remission	52/88(59.1 %)	51/98(52.0 %)	0.334
**Proteinuria remission rates at endpoint**			
24hUPE(g/24 h)	0.23(0.10,2.67)	0.75(0.14,3.74)	0.098
Complete remission	54(54.5 %)	45(42.9 %)	0.095
Overall remission	73(73.7 %)	73(69.5 %)	0.505
**Renal outcomes**			
ESRD	7(7.1 %)	4(3.8 %)	0.303
primary outcome^a^	25(25.3 %)	21(20.0 %)	0.370

*GC* glucocorticoid; *CTX *cyclophosphamide; *CNI* calcineurin inhibitor; *IST* immunosuppressive therapy; *24hUPE* 24 h urinary protein excretion; *ESRD* end-stage renal disease. ^a^Primary outcome was worsening of the renal function, defined as a 30 % or more decrease in eGFR or ESRD (eGFR < 15ml/min/1.73m2)

There was no significant difference for the 24hUTP, complete proteinuria remission rates and overall proteinuria remission rates at 6th month after induction therapy and at the end of follow-up (Table 3).

### Primary outcome

**Fig. 2:**
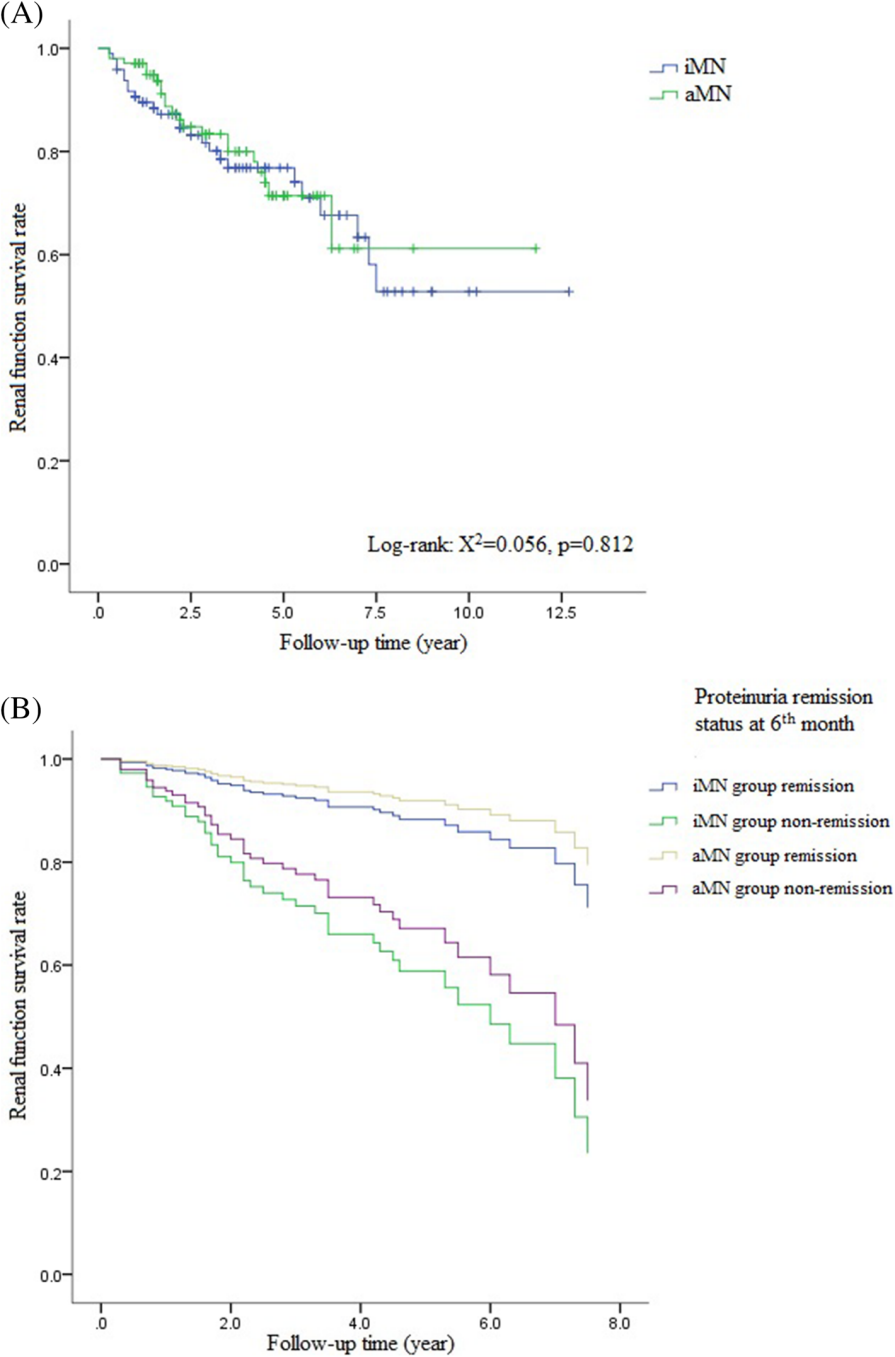
**a** Survival curves of iMN and aMN groups. **b** Effect of proteinuria remission status at 6th month on renal outcomes of iMN and aMN patients.

The primary outcome was worsening of the renal function, defined as a 30 % or more decrease in eGFR or ESRD (eGFR < 15ml/min/1.73m^2^). During follow-up, 7 iMN (7.1 %) and 4 aMN (3.8 %) patients developed ESRD (*p* = 0.303). The primary outcomes occurred in 25 (25.3 %) iMN and 21 (20.0 %) aMN patients during follow-up (*p* = 0.370). Kaplan–Meier analysis (Fig. 2 a) found no significant difference between groups (*X*^2^ = 0.056, *p* = 0.812). Multivariate COX regression model 1 (Table 4) showed that after baseline demographics, laboratory values and proteinuria remission status at 6th month were adjusted, aMN had a similar renal outcome compared with iMN, the HR of primary outcome was 0.735 (95 % CI 0.360 ~ 1.503, *p* = 0.399). Model 2 showed that proteinuria non-remission at 6th month was an independent risk factor of primary outcome in both iMN (HR = 4.248, 95 % CI 1.485 ~ 12.151, *P* = 0.007) and aMN group (HR = 3.194, 95 % CI 1.094 ~ 9.324, *P* = 0.034) (Fig. 2b).

**Table 4:** COX regression of worsening renal function

	Worsening of the renal function								
	Univariate COX regression			Multivariate COX regression(model 1)			Multivariate COX regression(model 2)		
	HR	95 % CI	*p* value	HR	95 % CI	*p* value	HR	95 % CI	*p* value
aMN^1^	0.931	0.515 ~ 1.683	0.813	0.735	0.360 ~ 1.503	0.399			
Age ^1,2^	1.019	0.997 ~ 1.041	0.086	1.021	0.976 ~ 1.068	0.365	1.020	0.975 ~ 1.068	0.387
Male gender^1,2^	1.620	0.852 ~ 3.080	0.141	0.505	0.161 ~ 1.584	0.241	1.986	0.632 ~ 6.240	0.240
Systolic pressure^1,2^	1.014	0.999 ~ 1.029	0.068	1.011	0.992 ~ 1.029	0.255	1.011	0.992 ~ 1.029	0.251
Diastolic pressure ^1,2^	1.006	0.980 ~ 1.033	0.644						
Smoking ^1,2^	1.900	1.062 ~ 3.397	0.030	1.590	0.715 ~ 3.537	0.256	1.594	0.715 ~ 3.552	0.254
Microscopic hematuria^1,2^	1.000	0.997 ~ 1.002	0.820						
Urinary protein excretion at baseline^1,2^	1.088	1.031 ~ 1.149	0.002	1.090	1.018 ~ 1.167	0.014	1.088	1.013 ~ 1.169	0.021
Serum creatinine^1,2^	1.009	1.002 ~ 1.017	0.008	1.011	0.983 ~ 1.039	0.448	1.011	0.983 ~ 1.039	0.452
Urea ^1,2^	1.156	1.056 ~ 1.266	0.002	1.014	0.857 ~ 1.199	0.870	1.014	0.857 ~ 1.200	0.870
eGFR^1,2^	0.986	0.975 ~ 0.997	0.013	1.017	0.972 ~ 1.064	0.466	1.017	0.972 ~ 1.063	0.473
Uric acid ^1,2^	1.004	1.002 ~ 1.007	0.001	1.003	1.000 ~ 1.006	0.077	1.003	1.000 ~ 1.006	0.077
Cholesterol^1,2^	1.030	0.913 ~ 1.161	0.635						
Triglyceride ^1,2^	1.039	0.946 ~ 1.142	0.421						
LDL-C^1,2^	0.943	0.818 ~ 1.087	0.421						
HDL-C ^1,2^	0.650	0.305 ~ 1.386	0.265						
Albumin ^1,2^	0.975	0.921 ~ 1.031	0.371						
Anti-PLA2R antibody concentration^1,2^	0.995	0.988 ~ 1.003	0.202						
Proteinuria non-remission at 6th month^1^	4.016	2.009 ~ 8.028	< 0.001	3.571	1.741 ~ 7.325	0.001			
Proteinuria remission at 6th month of iMN^2,a^	/		0.002	/			/		0.005
Proteinuria non-remission at 6th month of iMN^2^	3.333	1.399 ~ 7.945	0.007				4.248	1.485 ~ 12.151	0.007
Proteinuria remission at 6th month of aMN^2^	0.538	0.144 ~ 2.088	0.356				0.675	0.155 ~ 2.942	0.601
Proteinuria non-remission at 6th month of aMN^2^	3.210	1.362 ~ 7.565	0.008				3.194	1.094 ~ 9.324	0.034

1: included in Model 1, 2: included in Model 2,^a^As a reference

The univariate COX regression (Table 4) showed that older age, higher systolic blood pressure, smoking, higher baseline 24hUPE, creatinine, urea UA, eGFR level and proteinuria non-remission at 6th month were associated with the primary end point. Multivariate COX regression (both model 1 and 2) showed that baseline urinary protein (HR = 1.088, 95 %CI 1.013 ~ 1.169, *p* = 0.021) were independently associated with primary outcomes.

## Discussion

In our single center cohort, the rate of primary endpoint of aMN was similar with that of iMN after demographics, baseline laboratory values and proteinuria remission status at 6th month were adjusted. We also found that the proteinuria remission rates were similar between iMN and aMN group, and that non-remission at 6th month was an independent risk factor of primary endpoint, in both iMN and aMN group. This suggests that atypical MN is probably just a pathologic variant of primary MN, and not a distinct clinical-pathologic entity.

Our study was the first to summarize and compare the characteristics of treatment outcomes and renal prognosis between iMN patients and aMN patients. This new type of MN was called aMN, lupus-like MN or “full-house” MN, was characterized by cells proliferation, mesangial, subendothelial and subepithelial immune deposits, and “full-house” immunofluorescence staining for IgG, IgM, IgA, C3 and C1q[7, 8, 11] in most patients.

Wang et al[12] compared clinical features of 55 patients with aMN and 135 patients with iMN, and found no differences in sex, age, clinical manifestations, the levels of blood creatinine, 24hUPE, and the levels of serum IgA, IgG, IgM, and C3. Sam et al[9] reviewed and compared the baseline of iMN, membranous lupus nephritis, and ‘‘lupus-like’’ MN, discovered that patients with iMN were significantly older than patients with membranous lupus nephritis or ‘‘lupus-like’’ MN (46 versus 37 versus 38 years, respectively, p = 0.001), patients with ‘‘lupus-like’’ MN had proteinuria somewhere in between the other two groups (9.8 versus 4.2 versus 7.4 g/d, respectively, p = 0.001), with no significant differences in creatinine (p = 0.26). In our center, the average age of iMN patients was significantly older than aMN patients (56.1 versus 47.2 years), the baseline eGFR level of iMN patients was lower than aMN patients (90.59 versus 97.75 ml/min/1.73m^2^), the blood IgG level of iMN patients was higher than aMN patients (7.70 versus 6.85 g/L), while there were no significant differences in 24hUPE, creatinine, blood lipid and other immunological indicators between the two groups, the differences between these centers may be related to the ethnicity and geography of the population.

Renal prognoses of aMN patients reported by different centers were different. Sam et al[9]reported the average proteinuria after 3.5 years of follow-up was 5.7, 1.7, and 3.1 g/d, respectively, in iMN, membranous lupus nephritis, and ‘‘lupus-like’’ MN patients, showing statistically significant difference between them (*p* = 0.004), and at the end of follow-up, eleven of 39 (28 %) in iMN, two of 36 (6 %) in membranous lupus nephritis, and three of 23 (13 %) in “lupus-like” MN progressed to end-stage renal disease and dialysis commenced. However, Rijnink et al[13] discovered that there was no significant difference in the prognosis of non-lupus “full house” MN and lupus nephritis type V, but the former was an independent risk factor for ESRD compared with lupus nephritis type III/IV ± V (HR 5.31, 95 % CI 1.47–19.24). In our study, the 24hUPE, complete proteinuria remission rates and overall proteinuria remission rates and renal outcomes were similar in two groups, there was no difference in the incidence of ESRD between iMN and aMN patients (*p* = 0.303). The difference in proteinuria remission and incidence of ESRD between different centers may due to the fact that there is no uniform recommendation for treatment of aMN, some patients were treated more often with prednisone, cyclosporine, and cyclophosphamide, whereas otherpatients received more prednisone, mycophenolate, and azathioprine, the jury is still out on which immunosuppressant is more effective in patients with aMN.

As of now, we know that 70 %~80 % of primary MN is related to PLA2R[14] while an additional 1 %~5 % are associated with THSD7A[15]. Recently, using laser microdissection of glomeruli and mass spectrometry analysis of the proteins in the biopsies of patients with MN, Sethi et al. discovered another two antigens, exostosin[16], associated with autoimmune etiologies of the disease, and NELL-1, as a distinct cause of primary MN[17] or malignancy-associated MN[18]. However, there have been few reports of antigens/antibodies to aMN, previous study in our center suggested no difference in anti-PLA2R antibody positive rates between aMN and iMN patients (57.4 % vs. 48.1 %, p = 0.168)[7], which suggests that aMN and iMN may be the same disease in essence. Cytokine such as interleukin (IL)10 is responsible for the control of immune tolerance, but the overexpression of IL-10 interferes with activation, expansion and differentiation of B-cell, additionally, triggers mesangial cell expansion which was probably connected to impaired cell-mediated immunity in iMN [19, 20], and treatment against cytokines may improve the prognosis of iMN patients. Nevertheless, Caza et al. [18] compared the histopathologic parameters of NELL1-associated, PLA2R-associated, and THSD7A-associated membranous nephropathy, found that the occurrence rates of “full house” immunofluorescence were both less than 1 %, which suggests that genes, race, and countries and region may be involved. The pathogenesis and progression process of aMN patients may be more complex than that of iMN.

Besides, there were 3 patients in iMN group and 4 patients in aMN group developed SLE during follow-up, which were excluded in our study, and there had no changes on the results if these cases were included. Even so, we still need for long term follow-up to exclude incipient SLE, which may present with MN prior to the onset of serologic abnormalities and other clinical manifestations.

There are still some limitations to our study. Firstly, this is a retrospective and a single-center cohort study with a medium-sized study population, and there were some biases. Secondly, we regret that we were not able to test the PLA2R and the newest antigens on biopsy retrospectively yet, due to the large number of cases in this study, the workload of re-pathological sections is heavy, and further consultations are under way with the department of pathology. Finally, our study currently lacks detailed data on treatment, changes in levels of anti-PLA2R antibodies during follow-up, and mortality data.

## Conclusions

In our single center cohort, the overall proteinuria remission rates and renal outcomes were similar in iMN and aMN patients, suggesting that atypical MN is probably a pathologic variant of primary MN, but we still need for long term follow-up to confirm this, and further studies are needed to investigate the pathogenesis of aMN.

## Data Availability

The datasets used and analysed in the current study are available from the corresponding authors on reasonable request.
